# Urban-rural difference in satisfaction with primary healthcare services in Ghana

**DOI:** 10.1186/s12913-017-2745-7

**Published:** 2017-11-25

**Authors:** Sanni Yaya, Ghose Bishwajit, Michael Ekholuenetale, Vaibhav Shah, Bernard Kadio, Ogochukwu Udenigwe

**Affiliations:** 1 School of International Development and Global Studies, Faculty of Social Sciences, University of Ottawa, Ottawa, ON Canada; 2 School of Medicine and Health Management, Tongji Medical College, Huazhong University of Science and Technology, Wuhan, Hubei China; 3 The Women’s Health and Action Research Centre, Benin City, Nigeria; 4 Interdisciplinary School Health Sciences, University of Ottawa, Ottawa, ON Canada; 5 Faculty of Health Sciences, University of Ottawa, Ottawa, ON Canada

**Keywords:** Ghana, Patient satisfaction, Women, Urban-rural difference, Primary healthcare services

## Abstract

**Background:**

Understanding regional variation in patient satisfaction about healthcare systems (PHCs) on the quality of services provided is instrumental to improving quality and developing a patient-centered healthcare system by making it more responsive especially to the cultural aspects of health demands of a population. Reaching to the innovative National Health Insurance Scheme (NHIS) in Ghana, surpassing several reforms in healthcare financing has been a milestone. However, the focus of NHIS is on the demand side of healthcare delivery. Studies focusing on the supply side of healthcare delivery, particularly the quality of service as perceived by the consumers are required. A growing number of studies have focused on regional differences of patient satisfaction in developed countries, however little research has been conducted concerning patient satisfaction in resource-poor settings like in Ghana. This study was therefore dedicated to examining the variation in satisfaction across rural and urban women in Ghana.

**Methods:**

Data for the present study were obtained from the latest demographic and health survey in Ghana (GDHS 2014). Participants were 3576 women aged between 15 and 49 years living in non-institutional settings in Ghana. Summary statistics in percentages was used to present respondents’ demographic, socioeconomic characteristics. Chi-square test was used to find association between urban-rural differentials with socio-economic variables. Multiple logistic regression was performed to measure the association of being satisfied with primary healthcare services with study variables. Model fitness was tested by pseudo *R* ^2^. Statistical significance was set at *p* < 0.05.

**Results:**

The findings in this study revealed that about 57.1% were satisfied with primary health care services. The urban and rural areas reported 57.6 and 56.6% respectively which showed no statistically significant difference (z = 0.64; *p* = 0.523; 95%CI: -0.022, 0.043). Bivariate analysis showed that region, highest level of education, wealth index and type of facility were significantly associated with location of residence (urban-rural areas). After adjusting for confounding variables using logistic regression, geographical location became a key factor of satisfaction with primary healthcare services by location of residence. In urban areas, respondents from Greater Accra had 64% increase in the level of satisfaction when compared to those in Western region (OR = 1.64; 95CI: 1.09–2.47), Upper East had 75% increase in satisfaction compared to Western region (OR = 1.75; 95%CI: 1.08–2.84), Northern had an estimated 44% reduction in satisfaction when compared to Western region (OR = 0.56; 95%CI: 0.34–0.92). However, rural areas in Central, Volta, Eastern, Ashanti, Brong Aghafo, Northern and Upper West region had 51, 81, 69, 46, 62, 75 and 61% reduction respectively in the level of satisfaction when compared to Western region.

**Conclusions:**

Patient satisfaction is an important indicator of health outcomes. Quality of care and measuring level of patient satisfaction has been found to be the most useful tool to predict utilization and compliance. In fact, satisfied patients are more likely than unsatisfied ones to continue using health care services. Our results suggest that policymakers need to better understand the determinants of satisfaction with the health system and how different socio-demographic groups perceive satisfaction with healthcare services so as to address health inequalities between urban and rural areas within the same country.

## Background

Primary healthcare systems (PHCs) are widely recognised as the main foundation for every health delivery system. Since the declaration of Alma Ata in 1978, there has been a growing emphasis of the capacity and role of PHS in meeting the public health and well-being related goals of communities in a manner optimised for the local socioeconomic, cultural and political environment and preserving the fundamental human rights of the population at the same time [1]. PHCs is also considered a key to promoting health equity, ensuring access to essential care and thus promotion of universal health coverage (UHC) which is a priority focus for WHO and for the member states. Expert research evidences support the fact that UHC is not achievable through the hospital-oriented health delivery system and thus unable to meet the vision of Alma Ata. Despite these developments, PHCs fail to receive proper policy attention especially in the developing nations in Asia and Africa. Some common scenarios in PHCs include inadequate infrastructure development, shortage of skilled health workers, dissatisfaction about remuneration and professional status of health workers and lack of administrative transparency [1], which ultimately lead to reduction in service quality, lack of trust, worsening patient-physician relation and low care seeking behaviour [2]. Indeed, PHCs in developing countries are faced with multifaceted problems, solutions to which will require developing context specific strategies for dealing with both the service provider’s and consumers’ needs that may vary across sociocultural and geographic parameters. In light of this context, we conducted the present study among adult women in Ghana to gain a better understanding of their views regarding various aspects of PHCs in the country.

Healthcare financing in Ghana underwent several reforms from completely free services to the introduction of ‘token user fees’ and further to the introduction of fixed fees for all medical procedures done before the diagnosis of a given ailment. There was yet another amendment of charging the cost of drugs to the patients, which however resulted in inequitable distribution of drugs to the healthcare facilities thus engendering the “cash and carry system”. Despite the provision of exemptions to selected classes, issues with the cash and carry system like delay in seeking treatment for the poor and inadequate supply of prescription drugs led to the introduction of the National Health Insurance Scheme (NHIS) in 2005 [3]. The NHIS defies a system dependent on user fees and instead focuses on prepayment and risk pooling, a system of worldwide focus aimed at “eliminating financial barriers to healthcare utilization” [4, 5].

However, the NHIS focuses on the demand side factors without considering the supply side factors of the provision of healthcare services. These might result in a vicious cycle involving dissatisfaction for the insured and discouraging new members to be insured, ultimately resulting in the reduction of the demand for healthcare service utilization [5]. It is in this vein that studies focusing on the supply side factors, such as the quality and users’ perception of healthcare services, are required. Such studies will provide “continual feedback” toward the improvement of NHIS and reduce indirect costs of access to healthcare services. This is especially true for resource poor settings like Ghana [6]. In this vein, the current study focuses on the key factors affecting the levels of patient satisfaction and the current trend in patient satisfaction with the primary healthcare services of Ghana.

It should be noted, however, that patient satisfaction is one of the factors determining the quality of healthcare services. Again, the factors affecting the perception of quality primary healthcare services depend on the healthcare delivery set up. As will be seen in the foregoing discussion, many studies have been conducted to associate the factors determining patient satisfaction. However, these studies have been conducted in developed countries and less so in the resource poor settings like Ghana.

One of the studies conducted in Kuwait, to assess the factors behind patient satisfaction, for example, concluded that the communication time between the patient and physician was inadequate and that the patient would prefer attending emergency care services rather than primary healthcare clinics. The quality of communication between the physician and patient was also negatively rated for the primary healthcare clinics [7]. Another study conducted at Delhi, India found positive patient satisfaction at the primary healthcare centers and the factors underlying the positive perception were ease of access, cooperative health personnel, less waiting time and low cost [8].

Another study conducted in one of the remote islands of UK concluded that issues of access persist despite several policy initiatives, these issues might be resolved by inculcating the local priorities, expectations and demographic factors in future policies [9]. Moreover, another study in Israel attempted to track the quality of healthcare services by examining the informal complaints to the front-end employees. The study was inconclusive in terms of the findings and suggested further research to assess the quality of healthcare services at the primary healthcare level [10]. In the same vein, a study conducted at Riyadh, Saudi Arabia drew the rationale relating patient satisfaction to health-related behaviors like patient compliance, appointment keeping and use of medical services. These further highlight the importance of studying the levels of patient satisfaction at the primary healthcare level. The concept of satisfaction was defined as the "extent to which an individual's expectations compares with the experience(s)" and the conclusion focused on the importance of waiting time between registration and consultation for the patient as having an impact on the overall patient satisfaction [11].

It should be noted that these studies are conducted in diverse but relatively developed country settings unlike resource poor settings like Ghana. Again, the intricacy of the concept of “satisfaction” should also be considered. The lack of a standard definition of patient satisfaction, more so in the absence of comparative studies, relegates the importance of such studies to oblivion. The authors of the current study believe that it is true that the construct of “satisfaction” lacks clarity of a standard definition and is one of the limitations of interpreting such studies. However, these studies remain a guide toward policy implications for the betterment of primary healthcare services and their utilization. In the absence of a standard definition of patient satisfaction, the spectrum of factors that could be associated with it remains wide and unclear. For example, in the above studies, factors ranging from demographic variables to even factors such as the job satisfaction of the healthcare employees have been found to be associated with patient satisfaction.

In Ghana, health care is largely provided by the government and most times administered by the Ministry of Health and Ghana Health Services. The healthcare system is divided into 5 levels of providers, which include the health posts, which are foremost level, the primary care for rural areas, health centers and clinics, district hospitals, regional hospitals and tertiary hospitals. Primary health care (PHC) is regarded as a vital tool in achieving universal health coverage (UHC). From the Alma Ata declaration in 1978, many countries have adopted the method of improving PHC to increase effective health service delivery [12]. In developing regions such as sub-Sahara Africa (SSA), primary health care helps to fill the inequity and inequality in health care services among the vulnerable and most-at-risk populations. Evidence reveals that lack of efficiency across health facilities is common in developing countries such as Ghana [13, 14]. In Ghana, PHCs and Community Health Improvement Service (CHIPS) located at the lowest level of the health care system are fashioned to meet the basic needs of the population specifically in rural areas. Therefore, improving the quality of services at the foundation is paramount to achieving PHC objectives in Ghana. Sadly, ineffectiveness at this level which can be measured through patients’ satisfaction of health care received have not been sufficiently researched, despite that these should serve as the entry point of treatment in the health care system.

The current study however, highlights some of the factors, although not an exhaustive list, which might be associated with overall patient satisfaction. These findings are expected to serve as a guide for future research and underline the importance of continual feedback to policy decisions as has been noted above. Accordingly, satisfaction on 13 aspects incorporating arenas of wait times, staff attitudes, logistic support and ease of access was assessed against possible factors such as economic status, age, educational attainment and region of residence such as urban or rural set up.

## Methods

### Survey and sampling techniques

Data for the present study were obtained from the latest demographic and health survey in Ghana (GDHS, 2014). The primary objective of the survey was to generate recent reliable information on fertility, family planning, infant and child mortality, maternal and child health, and nutrition. This information will enhance informed policy decisions and will be used for planning, monitoring, and evaluating programs related to reproductive health and health in general. The survey was implemented by the Ghana Statistical Service (GSS), the Ghana Health Service (GHS), and the National Public Health Reference Laboratory (NPHRL) of the GHS as part of the International Demographic and Health Survey program known as MEASURE DHS, which is currently active in 90 countries. The survey was conducted under the auspices of the United States Agency for International Development (USAID) with the technical assistance of ICF International, based in the USA. The Demographic and Health Surveys (DHSs) are free, public datasets, though researchers must register with MEASURE DHS and submit a request before access to DHS data is granted. This data request system ensures that all users understand and agree to basic data usage ethics standards.

The survey lasted from early September to mid-December of 2014. Sampling technique involved a two-stage clustering encompassing both urban and rural areas across all ten administrative regions in the country. The first stage involved selecting clusters which are collections of enumeration areas (EAs). A total of 427 clusters were selected (216 in urban areas and 211 in rural areas). In the second stage, households were selected systematically from each EAs. A total of 12,831 households were selected for the survey and 11,835 households were finally interviewed successfully with a response rate of 99%. Further details are provided in the final report of the Ghana DHS 2014 report (GDHS 2014).

### Variables selection and measurement

The explanatory variables of primary interest were economic status, whereas patient satisfaction on various aspects of healthcare services in relation to area of residence (Rural and Urban areas), was entered as a dependent variable.

A set of 13 items pertinent to the quality assessment of PHCs were extracted from the GDHS data set. The participants were inquired about their satisfaction on the following components to which they could answer as either YES or NO: 1) Satisfaction with the time to wait for your turn, 2) Satisfaction with the time spent in the consulting/examination room, 3) Satisfaction with the time to wait for tests to be performed, 4) Satisfaction with the time to wait for test results, 5) Satisfaction with the time at pharmacy/dispensary, 6) Satisfaction with staff when they listened to the respondent, 7) Satisfaction with staff when they explained what was wanted, 8) Satisfaction with staff when they gave advice on treatment, 9) Satisfaction with the cleanliness of the facility, 10) Satisfaction with the easiness of finding where to go, 11) Satisfaction with comfort and safety while waiting, 12) Satisfaction with privacy during the examination, 13) Satisfaction with confidentiality and protection of personal information. The scoring procedure involved summing the 13 items measuring satisfaction for a respondent to generate total satisfaction level. The mean was obtained and the variable was dichotomized to “satisfied” if a respondent scored at least the mean or “not satisfied” if a respondent scored below the mean respectively.

### Covariates

Several covariates were included based on their relevance to the outcome variable: age (years) of respondents which are grouped in the interval; 15–19, 20–24, 25–29, 30–34, 35–39, 40–44 and 45–49.Geographical regions include; Western, Central, Greater Accra, Volta, Eastern, Ashanti, Brong Ahafo, Northern, Upper East and Upper West.

In addition, educational attainment was measured as No education, Primary, Secondary and Higher.The wealth status was measure as: poorest, poorer, middle, richer and richest.

Calculation of Wealth status: DHS provide no direct information on personal income; however, DHS employs a special technique to measure household wealth index and classify them into five groups: richest, richer, middle, poorer, and poorest. DHS programs employ wealth index as a proxy indicator for personal income status which is representative of an individual’s ability to afford personal healthcare needs. The process involves assigning wealth scores to household possessions e.g. floor, wall and roof material; type of cooking fuel; access to potable water and sanitation, ownership of radio, TV, refrigerator, motorcycle and others. Scoring is performed by principal components analysis, and based on their weighted wealth scores, households fall into five wealth quintiles ranging from poorest to richest. Measurement of wealth index is explained in detail elsewhere [1].

Educational attainment: Based on total years of completion of formal education, the following categories were used: No education, Primary, Secondary, and Higher.

### Ethics statement

Before each interview, all participants gave informed consent to take part in the survey. The DHS program maintains strict standards for ensuring data anonymity and protecting the privacy of all participants. ICF International ensures that the survey complies with the U.S. Department of Health and Human Services regulations for the protection of human subjects, whilst the host country ensures that the survey complies with local laws and norms. Further approval for this study was not required since the data is secondary and is available in the public domain. More details regarding DHS data and ethical standards are available at: https://dhsprogram.com/What-We-Do/Protecting-the-Privacy-of-DHS-Survey-Respondents.cfm.

### Data analysis

Summary statistics in percentages was used to present respondents’ demographic and socioeconomic characteristics. Chi-square test was used to find association between urban-rural differentials with socio-economic variables. Multiple logistic regression was performed to measure the association of being satisfied with primary healthcare services with study variables. Model fitness was tested by pseudo R^2^. Statistical significance was set at 95% confidence interval. Data were analyzed using STATA (StataCorp, College Station, TX, USA) version 12 and SPSS version 21.

## Results

Results from Table 1 indicated that the distribution of respondents by urban and rural location had no difference across the age categories (χ^2^ = 6.73; *p* = 0.347). However, region, level of education, wealth index and type of health facility used were associated with residence of respondents. In the GDHS 2014 data collection, region of respondents had varied frequency of representation across urban and rural areas. In the entire sample, more than half of the respondents (50.6%) had secondary level of education and nearly a quarter (24.8%) had no education; 17.1% of respondents had primary level of education and 7.5% had higher levels of education. The differentials of urban and rural residence revealed that about 71.1% of respondents in urban areas had a minimum of secondary education, while only about 44% had the equivalent in rural areas. Poor educational attainment was therefore more prevalent in the rural areas.

**Table 1:** Socio-Demographic Characteristics of Respondents by Location

Characteristic	Urban *n* = 1838 (%)	Rural *n* = 1738 (%)	Total *n* = 3576 (%)	Statistical indices
Age (years)				
15–19	168 (9.1)	177 (10.2)	345 (9.6)	χ^2^ = 6.725 df = 6 *p* = 0.347
20–24	306 (16.6)	324 (18.6)	630 (17.6)	
25–29	391 (21.3)	357 (20.5)	748 (20.9)	
30–34	338 (18.4)	292 (16.8)	630 (17.6)	
35–39	289 (15.7)	254 (14.6)	543 (15.2)	
40–44	201 (10.9)	179 (10.3)	380 (10.6)	
45–49	145 (7.9)	155 (8.9)	300 (8.4)	
Region				
Western	157 (8.5)	167 (9.6)	324 (9.1)	χ^2^ = 326.695 df = 9 *p* < 0.001*
Central	158 (8.6)	142 (8.2)	300 (8.4)	
Greater Accra	314 (17.1)	41 (2.4)	355 (9.9)	
Volta	129 (7.0)	190 (10.9)	319 (8.9)	
Eastern	174 (9.5)	167 (9.6)	341 (9.5)	
Ashanti	258 (14.0)	132 (7.6)	390 (10.9)	
Brong Ahafo	209 (11.4)	218 (12.5)	427 (11.9)	
Northern	154 (8.4)	200 (11.5)	354 (9.9)	
Upper East	178 (9.7)	244 (14.0)	422 (11.8)	
Upper West	107 (5.8)	237 (13.6)	344 (9.6)	
Highest Educational Level				
No formal education	271 (14.7)	616 (35.4)	887 (24.8)	χ^2^ = 336.980 df = 3 *p* < 0.001*
Primary	259 (14.1)	352 (20.3)	611 (17.1)	
Secondary	1085 (59.0)	726 (41.8)	1811 (50.6)	
Higher	223 (12.1)	44 (2.5)	267 (7.5)	
Wealth Index				
Poorest	120 (6.5)	753 (43.3)	873 (24.4)	χ^2^ = 1591.674 df = 4 *p* < 0.001*
Poorer	123 (6.7)	501 (28.8)	624 (17.4)	
Middle	377 (20.5)	346 (19.9)	723 (20.2)	
Richer	549 (29.9)	130 (7.5)	679 (19.0)	
Richest	669 (36.4)	8 (0.5)	677 (18.9)	
Type of facility				
Public/government	1378 (75.2)	1592 (91.8)	2970 (83.2)	χ^2^ = 175.643 df = 1 *p* < 0.001*
Private	455 (24.8)	143 (8.2)	598 (16.8)	

*p significant at *p* < 0.05

The distribution of respondents from the various wealth categories was again almost uniform with 24.4% being the maximum respondents from the poorest class and 17.4% being the minimum from the poorer class. From the report, the richer and richest categories accounted for 66.3% of respondents in urban areas, and only 8% from rural areas. Also, a prominent number of respondents (72.1%) from rural areas were in the poorest and poorer categories compared to 13.2% of respondents in urban areas. Details of the frequency distribution as per the different variables are shown in Table 1 below. The results also showed that utilization of public/government health facility was more prevalent in the rural areas (91.8%), while it was 75.2% in the urban areas. This showed that a private healthcare service is more utilized by urban residents than their rural counterparts.

Patient satisfaction was analyzed using 13 items grouped in three domains of service delivery which include; efficiency of service delivery, satisfaction with the staff including the consulting physician, and satisfaction with other logistics at the point of service. Efficiency was measured through four items; time to wait for your turn, time to wait for the tests performed, time to wait for the test results and time to wait at the pharmacy/dispensary with total satisfaction of 64.9, 80.3, 56.0 and 55.7% respectively. The disparities by place of residence (urban-rural) were presented in Table 2 below. More so, satisfaction with the staff was measured through four items; time spent in consultation, satisfaction when the staff listened to the respondents, satisfaction when the staff explained what was wanted and satisfaction when the staff gave advice on treatment. In addition, satisfaction on other logistics at the point of service was measured through five items; easiness of finding the facility, cleanliness of the facility, comfort and safety when waiting for service, privacy during examination and confidentiality and protection of personal information. Details of the items measuring satisfaction level among respondents are presented in Table 2.

**Table 2:** Percentage of items measuring patients’ satisfaction

Items	Urban	Rural	Total
Satisfaction of the time to wait for your turn	64.7	65.1	64.9
Satisfaction of the time spent in consulting/examination room	81.0	79.6	80.3
Satisfaction of the time to wait for tests to be performed	56.0	55.9	56.0
Satisfaction of the time to wait for test results	54.4	57.1	55.7
Satisfaction of the time at pharmacy/dispensary	71.1	69.3	70.2
Satisfaction of staff when they listened to the respondent	90.2	92.2	91.2
Satisfaction of staff when they explained what was wanted	84.6	88.4	86.4
Satisfaction of staff when they gave advice on treatment	83.3	86.4	84.8
Satisfaction of the cleanliness of the facility	92.3	92.4	92.4
Satisfaction of the easiness of finding where to go	91.6	91.3	91.5
Satisfaction of comfort and safety while waiting	87.9	87.7	87.8
Satisfaction of privacy during the examination	88.1	90.3	89.1
Satisfaction of confidentiality and protection of personal information	90.6	90.9	90.8

**Fig. 1:**
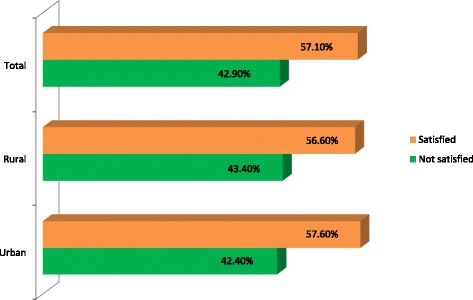
Satisfaction of service delivery by location

Differentials in the level of satisfaction showed that urban respondents reported 57.6% compared to 56.6% by their rural counterparts as shown in Fig. 1. In general, respondents showed that they were about 57.1% satisfied with the healthcare services in Ghana. This indicated that there is need for improvement in the quality of care which will also enhance positive health-care seeking behaviour of Ghanaians in general. The test of proportionality between urban and rural satisfaction level reported no statistically significant difference (z = 0.64; *p* = 0.523; 95%CI: -0.022, 0.043).

Multivariable logistic regression (results are shown in Table 3 above) was used to control for possible confounders in the model. After adjusting for covariates, geographical location (region) became a prominent explanatory variable of satisfaction with primary healthcare services across urban and rural locations. In the urban areas, respondents from Greater Accra were 1.64 times more likely to get satisfied when compared to those in Western region (OR = 1.64; 95CI: 1.09–2.47), Upper East were 1.75 times more likely to get satisfied when compared to Western region (OR = 1.75; 95%CI: 1.08–2.84), Northern had an estimated 44% reduction in satisfaction when compared to Western region (OR = 0.56; 95%CI: 0.34–0.92). However, rural areas in Central had 51% reduction in satisfaction, Volta had 81%, Eastern at 69%, Ashanti had 46%, Brong Aghafo reported 62%, Northern with 75% and Upper West region also had 61% reduction in the level of satisfaction when compared to Western region.

**Table 3:** Multivariate logistic regression analysis of service satisfaction in urban and rural Ghana

Factors	Urban		Rural	
	Odds ratio	*p*-value (95% CI)	Odds ratio	*p*-value (95% CI)
Region				
Western (ref.)	1.00		1.00	
Central	1.04	0.852(0.66–1.65)	0.49	0.004(0.30–0.80)*
Greater Accra	1.64	0.017(1.09–2.47)*	1.07	0.869(0.46–2.52)
Volta	1.05	0.858(0.64–1.71)	0.19	0.000(0.12–0.31)*
Eastern	0.75	0.201(0.48–1.17)	0.31	0.000(0.19–0.50)*
Ashanti	1.06	0.773(0.71–1.60)	0.54	0.017(0.32–0.89)*
Brong Ahafo	0.87	0.520(0.56–1.33)	0.38	0.000(0.24–0.61)*
Northern	0.56	0.021(0.34–0.92)*	0.25	0.000(0.15–0.41)*
Upper East	1.75	0.023(1.08–2.84)*	0.82	0.455(0.49–1.37)
Upper West	0.62	0.067(0.37–1.03)	0.39	0.000(0.24–0.65)*
Educational attainment				
No formal education(ref.)	1.00		1.00	
Primary	1.29	0.177 (0.89–1.88)	1.09	0.570 (0.81–1.46)
Secondary	0.91	0.559 (0.67–1.24)	0.99	0.933 (0.76–1.28)
Higher	1.12	0.599 (0.74–1.69)	0.74	0.427 (0.36–1.55)
Wealth index				
Poorest (ref.)	1.00		1.00	
Poorer	0.89	0.678 (0.53–1.52)	0.88	0.384 (0.67–1.17)
Middle	1.14	0.575 (0.72–1.80)	1.00	0.991 (0.71–1.42)
Richer	0.89	0.632 (0.56–1.41)	1.38	0.204 (0.84–2.29)
Richest	0.82	0.430 (0.51–1.34)	1.22	0.820 (0.22–6.87)
Type of facility				
Public/government (ref.)	1.00		1.00	
Private	1.99	0.000 (1.56–2.53)*	1.42	0.080 (0.96–2.09)

*p significant at *p* < 0.05; Urban-Pseudo *R* ^2^ = 0.04; Rural- Pseudo *R* ^2^ = 0.05

## Discussion

Healthcare financing has undergone several reforms in Ghana until the introduction of the National Health Insurance Scheme (NHIS), which strives for improvement in health delivery and utilization. The reforms were intended to reduce the inequity in access of healthcare services especially for the poor [15]. The NHIS focuses on prepayment and risk pooling to increase access to healthcare services. Patients’ satisfaction has been associated with specific factors, which encompass socio-economic, demographic and cultural factors. One of the studies noted that there are several factors like age, gender, marital status, education, family size and others, which play a role in the uptake of the NHIS [13]. However, the prime focus and effect of the scheme is on the demand side of the healthcare service delivery by making the services more affordable to the consumer. Several studies focusing on the demand side of the NHIS have been conducted [16–18]. However, the repercussion of affecting the demand side of healthcare delivery through the NHIS is an increase in the economic stress on the supply side. This has not been adequately studied. The supply side would include multiple factors such as available staff and logistics. Furthermore, the current trend is more toward patient centered care [19, 20]. To better understand patients’ level of satisfaction, the indicators used were overall satisfaction in waiting time, behaviour change communication by staff and satisfaction in confidentiality process.

Patient satisfaction is an important and commonly used indicator for measuring the quality of health care. In fact, practicing patient-centered care improves satisfaction rate and clinical outcomes. This study focused on studying the satisfaction of patients toward primary healthcare service delivery in Ghana. Items across the three domains; efficiency of service delivery, satisfaction with staff and satisfaction with other logistics at the point of service, were used to measure clients’ satisfaction. Contrary to our finding that showed no difference in satisfaction between urban and rural Ghana, a previous study has reported that satisfaction with local doctors and hospital services was higher in rural locations; rural patients were generally more satisfied with healthcare services compared to urban and suburban residents [21].

In the bivariate analysis, the association between place of residence (urban and rural) and socio-economic variables such as age, region, education, wealth index, type of facility were analyzed to examine possible statistical implications. There were significant statistical association based on the type of residence (urban or rural) and region, wealth index, level of educational attainment and type of healthcare facility accessed [22].

In terms of satisfaction with healthcare service delivery, after adjusting for educational attainment, type of facility and wealth index, certain geographical locations were significantly associated with satisfaction in health care services in urban and rural locations. These findings are consistent with previous studies of association with the type of residence, both without and after adjustment of factors such as region, educational attainment and wealth index [23, 24]. Another study also noted that a consumer friendly service and cordiality are paramount in providing primary health care services and increases satisfaction of the patients [25].

A previous study, although not entirely similar, related dissatisfaction of patients with the primary healthcare service buildings in general. Due to dearth of research studies related particularly to the differentials in place of residence and patients’ satisfaction related to primary healthcare services, further corroboration of studies was validated [26, 27]. There are significant differences in the overall health care assessment of rural populations as compared to urban populations.

This study has several strengths and limitations. Firstly, the dataset was large and included a broad range of indicators of service quality in Ghana. Data were analysed by carefully selected statistical methods that best suit the type of the data and were interpreted in light of the status quo in Ghana. However, the findings have limited external validity since the sample included only women. We also could not adjust for the health/diseases status of the participants as patients approaching with different complications might have differing opinions about the services they receive. Lastly, the data were cross-sectional and hence no causal relationship can be affirmed.

## Conclusion

In the present study we attempted to explore the regional variation in satisfaction about some key aspects of PHCs among Ghanaian women. Our results did not show any significant difference in the level of satisfaction of primary health care services between rural and urban residence. However, in the multivariate analysis, geographical region became a significant factor in satisfaction level between types of residence (urban-rural areas). The primary focus of the NHIS has remained on the demand side of the delivery of primary healthcare services. The current study is innovative in the sense of studying the levels of patient satisfaction across three domains, i.e. efficiency of healthcare delivery, satisfaction with staff attitudes and satisfaction toward other ethical procedures of primary healthcare service delivery. The study also attempted to find possible associations with type of facility, region, educational attainment and wealth index using urban-rural differentials.

There is no doubt patient satisfaction is an important indicator of health outcomes. Quality of care and measuring level of patient satisfaction has been found to be the most useful tool to predict utilization and compliance. In fact, studies showed that satisfied patients are more likely than unsatisfied ones to continue using health care services. Our results suggest that policymakers need to better understand the indicators of satisfaction with the health system and how different socio-demographic groups perceive satisfaction with healthcare services so as to address health inequalities between urban and rural areas within the same country.

## Abbreviations

DHS: Demographic and Health Surveys
EAs: Enumeration areas
GHS: Ghana Health Service
MDGs: Millennium Development Goals
MNCH: Maternal, newborn and child health
NHIS: National Health Insurance Scheme
